# Reasons for Tooth Removal in Adults: A Systematic Review

**DOI:** 10.1016/j.identj.2021.01.011

**Published:** 2021-02-26

**Authors:** Dyonne L.M. Broers, Leander Dubois, Jan de Lange, Naichuan Su, Ad de Jongh

**Affiliations:** a Department of Social Dentistry and Behavioral Sciences, Academic Centre for Dentistry Amsterdam, Amsterdam, The Netherlands; b Department of Oral and Maxillofacial Surgery, Amsterdam University Medical Centers, University of Amsterdam, Academic Centre for Dentistry Amsterdam, Amsterdam, The Netherlands; c Department of Oral and Maxillofacial Surgery, St Antonius Hospital, Nieuwegein, Utrecht, and Woerden, The Netherlands; d Department of Oral and Maxillofacial Surgery, Amsterdam University Medical Centers, University of Amsterdam, Academic Centre for Dentistry Amsterdam, Amsterdam, The Netherlands; e Department of Oral and Maxillofacial Surgery, Isala klinieken Zwolle, The Netherlands; f Department of Social Dentistry and Behavioral Sciences, Academic Centre for Dentistry Amsterdam, Amsterdam, The Netherlands; g Department of Social Dentistry and Behavioral Sciences, Academic Centre for Dentistry Amsterdam, Amsterdam, The Netherlands; h Research Department PSYTREC, Bilthoven, The Netherlands; i School of Health Sciences, Salford University, Salford, United Kingdom; j Institute of Health and Society, University of Worcester, Worcester, United Kingdom; k School of Psychology, Queen's University, Belfast, Northern Ireland

**Keywords:** Clinical decision-making, Motivation, Patient preference, Tooth extraction

## Abstract

**Objective:**

Most tooth extractions are performed for dental reasons, but there are also nondental and nonmedical reasons for extractions; these include psychological, financial, religious, and cultural reasons as well as simply granting a patient's request. This systematic review was performed to examine the proportion and range of indications associated with tooth removal in context of dental, nondental, and medical reasons.

**Methods:**

A search conducted using PubMed, Embase, and APA PsycINFO identified 6038 studies. Three studies (4396 extractions in total) could be included for the risk of bias assessment and qualitative data synthesis.

**Results:**

The reported indications for tooth extraction on dental and medical grounds included caries with the proportion of all extractions ranging from 36.0% to 55.3%, periodontitis from 24.8% to 38.1%, trauma from 0.8% to 4.4%, periapical disease from 7.3% to 19.1%, orthodontics from 2.5% to 7.2%, and other reasons from 4.5% to 9.2%. The proportion for patient requests ranged from 3.6% to 5.9%, but specific information regarding the actual reasons for extraction could not be determined.

**Conclusion:**

The results suggest that caries and periodontitis are the most common indications for tooth extraction and that studies to reliably estimate the incidence of nondental and nonmedical motivation for extraction are lacking. Given that the final decision on performing or refusing extractions, whether it be based on dental, nondental, or nonmedical reasons, largely rests with the dentist and oral surgeon, detailed guidelines are warranted.

## Introduction

Dental extractions are routine procedures performed by dentists all over the world. Estimates of the proportion of tooth extractions in the total amount of dental treatments varies by country and may differ between Western and non-Western countries. For example, a study among 17,784 Swiss patients, ages 15 to 74 years, showed that 5.4% of all dental treatments were extractions.^1^ A study from Brazil found that 10.2% (*n* = 161,812,852) of all dental treatments between 1998 and 2012 were dental extractions of permanent teeth.^2^ With regard to the indications for extractions, caries is generally described as the main indication.^3^, ^4^, ^5^, ^6^, ^7^, ^8^ Other indications are also reported such as periodontitis, endodontic problems, orthodontic considerations, failure of eruption, part of a prosthetic treatment plan,^9^^,^^10^ dental trauma, aesthetic, and other medical reasons that would justify treatment (eg, elimination of dental foci before immunotherapy or radiotherapy treatment). However, up-to-date overviews and estimates regarding the relative magnitude of reasons or indications for extractions in general are lacking, as well as reasons concerning period, cultural background, and region.

Occasionally patients request that their dentist or oral (and maxillofacial) surgeon remove their teeth based on dental fear or due to financial, religious, or cultural reasons. Although these requests may be incompatible with the “standard of care” provided by a good health care provider, the pressure on the practitioner to fulfil such requests can be high.^11^ To this end, professional organizations have drawn up different standards or rules of conduct, but none of these are specifically aimed at decisions regarding dental extractions (other than a third molar). It can also be argued that given that dental extraction is an irreversible treatment, it is essential that the decision-making process of the health care professional is carefully followed.^11^ Knowledge about how often these situations arise can help develop guidelines that support dentists and oral surgeons in delineating ethical and legal issues and guide them in decision-making and responding to extraction requests.

This systematic review assesses the main reasons for extractions for permanent teeth in adults. In line with this, we aim to determine potential differences in reasons for extractions over time and examine cultural or regional differences. A second aim of the present study is to derive an estimate of the incidence of requests for dental extraction of permanent teeth on nondental and nonmedical grounds and the proportion of dental extractions of permanent teeth on nondental and nonmedical grounds (eg, patients’ request) that were rejected.

## Methods

A systematic review was performed, following the guidelines of the Preferred Reporting Items Systematic Review and Meta-Analysis (PRISMA) statement and using the explanation and elaboration document.^12^ This review was registered in PROSPERO (registration number CRD42020184804). The search was conducted on November 6, 2020, in the following databases: PubMed, Embase, and APA PsycINFO. Table 1 shows the search terms and Supplementary file 1 shows the search protocol that were used. For additional searching, grey literature and hand searching (eg, snowballing) were used.

**Table 1:** Search terms.

tooth extraction, teeth extraction, dental extraction, tooth removal, teeth removal, tooth loss, adult, adolescent, aged, age factors, age distribution, permanent dentition, permanent teeth, reason, motive, cultural diversity, religious reasons, poverty, financial reasons, social values, dental caries, complications, DMF index, tooth fractures, impacted tooth, eruption problems, surgery, oral surgical procedures, preprosthetic, corrective methods, pericoronitis, periodontal disease, periodontitis, complications, orthodontics, root canal therapy, endodontic problems, dental trauma, edentulous, esthetic reasons, medical reasons, dental foci, dental anxiety, dental fear, psychology, mental competency, somatoform disorders, post-traumatic stress disorders request, rejection, refusal to treat, ethics, etiology, trends, statistics, numerical data, survey, epidemiology, therapy

### Inclusion and exclusion criteria

Patients included were 18 years and older, with a request for extraction of 1 or more permanent teeth. Only adults were included because interest was focussed on requests of patients themselves and not of their parents—because children up to the age of 18 are minors, a request for extraction may not only come from the child but also from the parent. The reason for inclusion of patients with extractions of permanent teeth was based on the fact that extraction of a permanent tooth is an irreversible treatment and deciduous teeth have permanent successors. Following this, third molars were excluded, given that in most cases these are not essential for functioning or aesthetics. There was no requirement as to the sample size. Only original studies published in English, German, French, and Dutch were selected. Animal studies were excluded. When there was a specific sample or population, for example those who need prosthetic or periodontal treatment, then that study was excluded. Studies in which a limited range of categories was used (eg, caries and periodontitis only) were also excluded because this makes interpretation difficult. When it was unclear from a full text whether deciduous teeth or third molars were included, then these studies were also excluded.

For assessing the quality of the studies, the risk of bias tool for prevalence studies adapted from Hoy et al^13^ was used. This instrument is easy to use and has a high interrater agreement. Each of the 9 questions can be answered with ‘yes’ (0 points) or ‘no’ (1 point) and added up to a maximum score of 9 points. For the summary assessment of risk of bias an overall score of 0-3 was used to indicate low risk, 4-6 moderate risk, and 7-9 high risk. Given that there was a lack of studies with low risk of bias we included studies with both moderate and high risk of bias.

### Retrieval and selection

The figure illustrates the screening and selection protocol flow diagram. The search terms were drawn up by 2 reviewers (DLMB, AdJ) and a librarian (Table 1). Duplicates were removed followed by a first screening on title and abstract by 2 reviewers independent of each other (DLMB, AdJ). Any deviations were discussed until full agreement was reached. Next, full texts were analysed from all the remaining studies by the 2 reviewers independently. Again here, differences were discussed and resolved when a consensus was reached. After the full-text screening, 2 reviewers (DLMB, LD) assessed the risk of bias. A final analysis of the included studies was carried out by 3 reviewers (DLMB, AdJ, LD).

**Fig:**
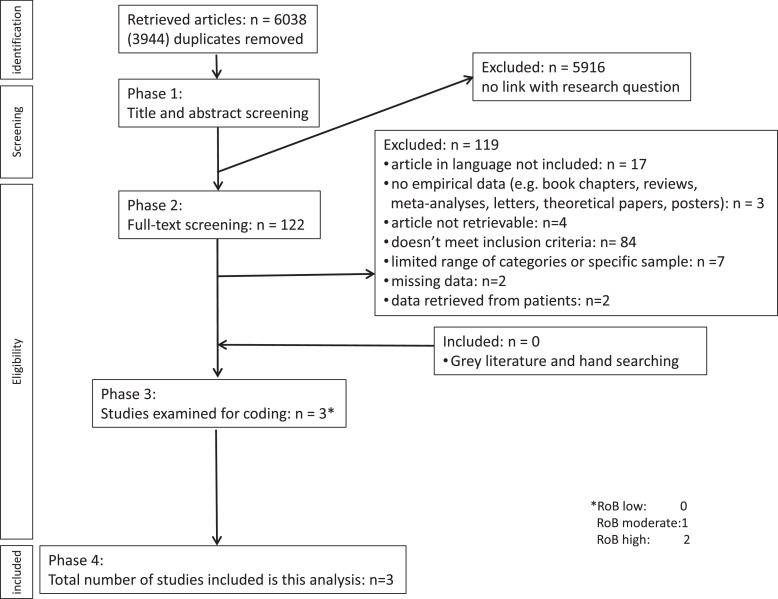
Selection protocol.

### Data extraction and synthesis

After having considered the risk of bias of the included studies (eg, the extent to which the included studies addressed the review objectives and made it possible to conduct a quality assessment in terms of numbers and type of teeth extractions), a narrative synthesis was conducted that included an investigation on the similarities and the differences between the findings of the different studies, as well as an exploration of the different reasons for teeth extraction identified in the included studies. We systematically considered the results from the included studies in terms of the number of studies, the reasons for extractions, and their results. Next, each of the included studies was described in terms of study design, setting, population, period, culture, and outcomes expressed as reasons for extractions and represented as proportions with 95% CI. Acquired data sets were transformed into a descriptive or possible statistical format to identify similarities between studies. Reasons for differences (ie, variability) in outcomes, study designs, populations, settings, cultures, nations, and patient characteristics were explored, and it was considered to what extent the results of the studies might have been affected by factors such as methodological differences between studies or variable characteristics of the populations (ie, heterogeneity between and within studies). These results were tabulated to identify patterns across the included studies and to explore the need for subgroup analyses.

## Results

Initially, 9982 studies were found: 4678 in PubMed, 5208 in Embase, and 96 in APA PsycINFO. After the removal of duplicates (3944), 6038 studies remained. Next, titles and abstracts were screened and 5916 studies were excluded, whereby 122 articles remained. Reference lists of these studies were also checked for relevant articles and unpublished manuscripts. No additional studies were found. After full-text screening, 17 articles were excluded because of language, 2 because it was a letter to the editor without data and 1 was excluded because it was a review instead of an original study. Four studies could not be retrieved, even after contacting the authors. For 2 studies, no contact information was available. In total, 83 articles were excluded because these did not meet the inclusion criteria. More specifically, studies that contained data about children (*n* = 54), mentioned no age range (*n* = 9), or noted full mouth extractions (*n* = 3), these were excluded because in the latter case it was impossible to determine the reasons for extraction per tooth. We also excluded studies in which third molars were included (*n* = 3). Four more studies did not fully meet our inclusion criteria, for instance, in terms of age range or the fact that there were doubts as to whether patients were younger than 18-years or third molars had been included in the data.^3^^,^^14^, ^15^, ^16^ Corresponding authors of the mentioned articles were contacted with a request to provide the raw data from their studies. One author indicated to no longer possess any raw data and because 3 authors did not respond to our query despite reminders on several email addresses, these studies were excluded. In 7 excluded articles. only a few reasons for extractions were mentioned or specific samples were used, 2 articles did not contain sufficient data to draw conclusions, and in 2 others the reasons for extraction were only given by the patient. Finally, 3 studies remained in which their full text was analysed and examined for risk of bias. Of all remaining studies, no study had a low risk of bias, 1 study had a moderate risk of bias,^17^ and in 2 studies the risk of bias was high.^18^^,^^19^ Accordingly, 3 studies remained for data synthesis yielding a total of 4396 extractions and 1896 patients that could be included. One study was performed between 2007 and 2010 in Greece,^17^ 1 study between 2004 and 2009 also in Greece,^18^ and 1 study in 1995 in Croatia.^19^ A summary of study characteristics are presented in Table 2.

**Table 2:** Study characteristics.

Reference	Study design	Sample characteristics	Age range (y); mean (y)	Data collection period	Country	Patients (n)	m/f (%)	Extractions (n)	Risk of bias*
Chrysanthakopoulos, 2011a	Prospective, cross-sectional (questionnaires and clinical examination)	Mean number of 20 natural teeth, with good general health, who attended a private practice	range 18-76, mean 44.6 ± 5.8	Aug 2007-July 2010	Greece	632	53.8:46.2	1688	6
Chrysanthakopoulos, 2011b	Prospective, cross-sectional (questionnaires and clinical examination)	Mean number of 20 natural teeth, with good general health, who attended a private practice	range 18-74, mean 42.6 ± 5.8	June 2004-May 2009	Greece	1018	55.0:45.0	2462	7
Plančak et al, 2004	Retrospective, cross-sectional (data from dental records)	Civilian population during war	range 18-59	1995	Croatia	246		246	7

*Risk of bias^13^: 0-3 low; 4-6 moderate; 7-9 high.

### Main dental reasons for extractions of permanent teeth

Three studies reported caries, periodontitis, and trauma as the reasons for extraction.^17^, ^18^, ^19^ The most common reason reported for extraction was caries with the proportions of 36.0% (95%CI, 33.8-38.3), 45.6% (95%CI, 43.7-47.6), and 55.3% (95%CI, 49.0-61.4), and periodontitis with the proportions of 24.8% (95%CI, 19.7-30.5), 32.1% (95%CI, 30.3-34.0), and 38.1% (95%CI, 35.8-40.4). Other categories were trauma with the proportions of 0.8% (95%CI, 0.1-2.7), 3.6% (95%CI, 2.7-4.5), and 4.4% (95%CI, 3.6-5.3). Two studies reported orthodontics and other reasons as the reasons for extraction.^17^^,^^18^ Another 2 studies reported periapical disease as the reason for extraction.^18^^,^^19^ Periapical disease had the proportions of 7.3% (95%CI, 6.3-8.4) and 19.1% (95%CI, 14.6-24.4). Orthodontics had the proportions of 2.5% (95%CI, 2.0-3.2) and 7.2% (95%CI, 6.1-8.5). Other reasons had the proportions of 4.5% (95%CI: 3.8-5.4) and 9.2% (95%CI, 7.9-10.7; Table 3).

**Table 3:** Indications for extraction (dental and medical).

Reference	Caries, % (95% CI)	Caries, *N*	Periodontitis, % (95% CI)	Periodontitis, *n*	Trauma, % (95% CI)	Trauma, *n*	Periapical disease,* % (95% CI)	Periapical disease, *n*	Orthodontics, % (95% CI)	Orthodontics, *n*	Other reasons, % (95% CI)	Other reasons, *n*
Chrysanthakopoulos, 2011a	36.0 (33.8%-38.3%)	608	38.1 (35.8%-40.4%)	643	3.6 (2.7%-4.5%)	60			7.2 (6.1%-8.5%)	122	9.2† (7.9%-10.7%)	156
Chrysanthakopoulos, 2011b	45.6 (43.7%-47.6%)	1123	32.1 (30.3%-34.0%)	790	4.4 (3.6%-5.3%)	108	7.3 (6.3%-8.4%)	180	2.5 (2.0%-3.2%)	62	4.5‡ (3.8%-5.4%)	111
Plančak et al, 2004	55.3 (49.0%-61.4%)	136	24.8 (19.7%-30.5%)	61	0.8 (0.1%-2.7%)	2	19.1 (14.6%-24.4%)	47				

⁎ Periapical disease, failed root canal treatment.

† Other reasons: impacted teeth, pericoronitis, unspecified reasons, etc.

‡ Other reasons: eg, impacted teeth, prosthetic indications.

### Potential differences in indications for extractions over time and per region (eg, culture, country)

A further breakdown regarding differences over time or different cultural contexts was impossible. For example, 2 out of 3 studies were performed in the same year and region.

### The probable incidence of a request for dental extraction of permanent teeth on nondental and nonmedical grounds

In 2 studies patient requests were reported.^17^^,^^18^ In 3.6% (95%CI, 2.9-4.4; *n* = 100) to 5.9% (95%CI, 4.9-7.1; *n* = 89) of the cases nondental and nonmedical reasons (ie, “patient requests”) were given as an explanation regarding teeth extractions, but these requests were not further specified. In 1 study, patient request was not mentioned.^19^

### The proportion of dental extractions of permanent teeth on nondental and nonmedical grounds that are likely to be rejected

None of the included studies reported information on dental extractions that were rejected; hence, addressing this research question could not be answered.

## Discussion

The results of this review suggest that caries and periodontitis are the main indications for dentists and oral (and maxillofacial) surgeons to perform dental extractions. Other frequent indications are trauma, periapical disease, orthodontics, and other reasons. This is generally in line with previous studies, including those who did not meet the inclusion criteria of the present review.^3^, ^4^, ^5^, ^6^, ^7^, ^8^, ^9^, ^10^ Although it was part of our aim to gain information on possible changes in indications given by dentists and oral (and maxillofacial) surgeons for extractions and how often patients ask their practitioner to have their teeth removed over time and per region, these questions could not be determined. It appeared that almost 5% of the extractions performed were requested by the patient themselves. Unfortunately, the motivation of the patient in case of patient request that contributed towards extraction could not be specified; this, of course, could be due to financial or cultural motives, but it is not inconceivable that psychological reasons such as dental fear may have played a role.

Perhaps the most interesting result of the present systematic review is that the issue of extractions that are carried out by patient request per se has never properly been investigated. In other words, on the one hand it could be argued that there is little information to report in this area, and on the other hand, it has been of pivotal importance to ascertain through this review that information about nondental and nonmedical reasons is completely missing. This is particularly remarkable given the legal and ethical aspects underlying treatment decision and the fact that a dental practitioner or oral (maxillofacial) surgeon should not simply remove 1 or more teeth without dental necessity. Even when it is a patient's strict request, the practitioner may be held liable for such a treatment when no pure dental necessity is present.^11^ It is also unfortunate that it was impossible to derive an estimate as to how often these requests are *rejected* because this issue has also never been addressed in the literature before. The same goes for the limited number of, and not well-defined, categories involving the indications for extractions in the studies that were included and analysed. For example, in the 3 studies that were examined, a wide variety of categories or no descriptions of categories at all were used. In 2 studies, the category “other reasons” included impacted teeth, pericoronitis, and unspecified reasons,^17^^,^^18^ whereas in 1 study the same category included impacted teeth, prosthetic, and other reasons.^19^ Furthermore, these studies are at odds with study design (eg, retrospective vs prospective, clinical study vs dental record), inclusion criteria (ie, at least 20 teeth), or circumstances (ie, during war). In addition, the risk of bias of the studies was generally moderate to high, whereby it is striking that retrospective dental record studies showed a greater risk of bias than prospective clinical studies or questionnaire studies.^13^

The present study has strengths and limitations. One of the strengths of our systematic review compared to previously conducted clinical studies is the fact that we excluded third molars and children. Only permanent teeth were included because deciduous teeth have a permanent successor. Removal of deciduous teeth, therefore, is less likely to result in long-term damage to function and aesthetics. The extraction of third molars was an exclusion criterion because removal has no or little effect on aesthetics or oral function. Thus, based on these considerations we could have obtained important insights but due to strict inclusion and exclusion criteria, only a limited number of studies could be included.

Thorough research revealing the actual reasons for extraction on nondental and nonmedical grounds on permanent dentition (other than third molars) in adults is essential (ie, in which cases such requests are honoured by extracting the tooth and what the argument is for whether to actually perform the extraction). Future studies with a low risk of bias should use multiple well-defined categories for nondental and nonmedical reasons for extraction. Based on the current results, our proposal would be to define the following dental categories: caries, periodontitis, trauma, periapical disease, orthodontics, prosthodontics, supernumerary teeth, and medical reasons (eg, tumours). Besides these listed categories we propose nondental and nonmedical categories including patient requests (ie, aesthetic, psychological, financial, religious, or cultural reasons). The category psychological reasons should include dental phobia although other relevant subcategories (eg, body dysmorphic disorder, posttraumatic stress disorder, bipolar and depressive disorders, schizophrenia spectrum and other psychotic disorders, somatic symptom and related disorders, and neurocognitive disorders) may also be needed. A complicating factor is that studies aimed at assessing reasons for extractions are notoriously difficult to carry out because the reasons given by the dentist and oral and maxillofacial surgeon frequently reflect treatment options (including their financial aspects) rather than diseases per se.

## Conclusion

In conclusion, the results of the present review show that caries and periodontitis are the most common indications for dental extraction in permanent teeth. Unfortunately, no conclusions can be drawn on extractions based on nondental and nonmedical grounds. Health care professionals have to adhere both to the principle of nonmaleficence and on the principle of patient autonomy. This can pose dilemmas when deciding whether or not to remove teeth.^11^ Given that the final decision about performing or refusing dental extractions, whether based on either dental, nondental, or nonmedical reasons, largely rests with the dentist and oral (and maxillofacial) surgeon. Guidelines as to how to deal with such requests are warranted.

## Appendix Supplementary materials

Supplementary file 1. Search strategy. Image, application 1

## Footnotes

Supplementary material associated with this article can be found, in the online version, at doi:10.1016/j.identj.2021.01.011.
