# Supplementary material for: Reasons for Tooth Removal in Adults: A Systematic Review
Source: Int Dent J. 2021 Feb 26;72(1):52–7. doi: 10.1016/j.identj.2021.01.011 (PMC9275356; doi:10.1016/j.identj.2021.01.011)
Supplement: Supplementary file 1 — Search strategy. [file mmc1.docx]

Supplementary file 1. Search strategy.

**Search date: November, 6, 2020**

| **Reasons for Tooth extraction** | | **Refusal Tooth extraction** |
| --- | --- | --- |
| **Database** | **Results** | **Results** |
| PubMed | 4283 | 395 |
| Embase.com | 4587 | 621 |
| APA PsycInfo | 89 | 7 |
| Total | 8959 | 1023 |
| After removal duplicates | 5350 | 688 |

PubMed History and Search Details November 6^th^ 2020

| **Search** | **Query** | **Results** |
| --- | --- | --- |
| #7 | #6 NOT (("Adolescent"[Mesh] OR "Child"[Mesh] OR "Infant"[Mesh] OR adolescen*[tiab] OR child*[tiab] OR schoolchild*[tiab] OR infant*[tiab] OR girl*[tiab] OR boy[tiab] OR boys[tiab] OR boyhood[tiab] OR teen[tiab] OR teens[tiab] OR teenager*[tiab] OR youth*[tiab] OR pediatr*[tiab] OR paediatr*[tiab] OR puber*[tiab]) NOT ("Adult"[Mesh] OR adult*[tiab] OR man[tiab] OR men[tiab] OR woman[tiab] OR women[tiab])) | 4,283 |
| #6 | #4 OR #5 | 5,064 |
| #5 | #1 AND #3 | 4,541 |
| #4 | #1 AND #2 | 700 |
| #3 | "reason*"[tiab] OR "motiv*"[tiab] OR "factor*"[tiab] | 3,960,374 |
| #2 | "Dentition, Permanent"[Mesh] OR "permanent teeth"[tiab] OR "secondary teeth"[tiab] OR "secondary tooth"[tiab] OR "adult teeth"[tiab] OR "adult tooth"[tiab] OR "dentes permanent*"[tiab] OR "permanent dentition"[tiab] OR "adult dentition"[tiab] OR "secondary dentition"[tiab] | 9,831 |
| #1 | "Tooth Extraction"[Mesh] OR "tooth extraction*"[tiab] OR "teeth extraction*"[tiab] OR "dental extraction*"[tiab] OR "tooth removal"[tiab] OR "teeth removal"[tiab] OR "tooth loss"[tiab] OR "molar extraction*"[tiab] OR "serial extraction"[tiab] | 28,014 |

Embase History and Search Details November 6^th^ 2020

| **Search** | **Query** | **Results** |
| --- | --- | --- |
| #8 | #7 NOT (('adolescent'/exp OR 'child'/exp OR adolescent*:ti,ab OR child*:ti,ab OR schoolchild*:ti,ab OR infant*:ti,ab OR girl*:ti,ab OR boy*:ti,ab OR teen:ti,ab OR teens:ti,ab OR teenager*:ti,ab OR youth*:ti,ab OR pediatr*:ti,ab OR paediatr*:ti,ab OR puber*:ti,ab ) NOT ('adult'/exp OR 'aged'/exp OR 'middle aged'/exp OR adult*:ti,ab OR man:ti,ab OR men:ti,ab OR woman:ti,ab OR women:ti,ab)) | 4,587 |
| #7 | #6 NOT (('adolescent'/exp OR 'child'/exp OR adolescent*:ti,ab OR child*:ti,ab OR schoolchild*:ti,ab OR infant*:ti,ab OR girl*:ti,ab OR boy*:ti,ab OR teen:ti,ab OR teens:ti,ab OR teenager*:ti,ab OR youth*:ti,ab OR pediatr*:ti,ab OR paediatr*:ti,ab OR puber*:ti,ab ) NOT ('adult'/exp OR 'aged'/exp OR 'middle aged'/exp OR adult*:ti,ab OR man:ti,ab OR men:ti,ab OR woman:ti,ab OR women:ti,ab)) | 5,351 |
| #6 | #4 OR #5 | 6, 225 |
| #5 | #1 AND #3 | 5,665 |
| #4 | #1 AND #2 | 762 |
| #3 | reason*:ti,ab OR motiv*:ti,ab OR factor*:ti,ab | 5,112,522 |
| #2 | 'secondary dentition'/exp OR 'permanent teeth':ti,ab OR 'secondary teeth':ti,ab OR 'secondary tooth':ti,ab OR 'adult teeth':ti,ab OR 'adult tooth':ti,ab OR 'dentes permanent*':ti,ab OR 'permanent dentition':ti,ab OR 'adult dentition':ti,ab OR 'secondary dentition':ti,ab | 9,519 |
| #1 | 'tooth extraction'/exp OR 'tooth extraction*':ti,ab OR 'teeth extraction*':ti,ab OR 'dental extraction*':ti,ab OR 'tooth removal':ti,ab OR 'teeth removal':ti,ab OR 'tooth loss':ti,ab OR 'molar extraction*':ti,ab OR 'serial extraction':ti,ab | 32,747 |

APA PsycInfo History and Search Details November 6^th^ 2020

| **Search** | **Query** | **Results** |
| --- | --- | --- |
| S8 | #6 NOT ((ZG ("adolescence (13-17 yrs)" OR "childhood (birth-12 yrs)" OR "infancy (2-23 mo)” OR "neonatal (birth-1 mo)" OR "preschool age (2-5 yrs)" OR "school age (6-12 yrs)") OR TI (adolescen* OR child* OR schoolchild* OR infant* OR girl* OR boy* OR teen OR teens OR teenager* OR youth* OR pediatr* OR paediatr* OR puber*) OR AB (adolescen* OR child* OR schoolchild* OR infant* OR girl* OR boy* OR teen OR teens OR teenager* OR youth* OR pediatr* OR paediatr* OR puber*)) NOT (ZG ("adulthood (18 yrs & older)" OR "aged (65 yrs & older)" OR "middle age (40-64 yrs)" OR "thirties (30-39 yrs)" OR "very old (85 yrs & older)") OR TI (adult* OR man OR men OR woman OR women) OR AB (adult* OR man OR men OR woman OR women))) | 89 |
| S6 | S4 OR S | 97 |
| S5 | S1 AND S3 | 95 |
| S4 | S1 AND S2 | 3 |
| S3 | DE "Motivation" OR TI (reason* OR motiv* OR factor*) OR AB (reason* OR motiv* OR factor*) | 998,948 |
| S2 | TI ("permanent teeth" OR "secondary teeth" OR "secondary tooth" OR "adult teeth" OR "adult tooth" OR "dentes permanents" OR "permanent dentition" OR "adult dentition" OR "secondary dentition") OR AB ("permanent teeth" OR "secondary teeth" OR "secondary tooth" OR "adult teeth" OR "adult tooth" OR "dentes permanents" OR "permanent dentition" OR "adult dentition" OR "secondary dentition") | 65 |
| S1 | TI (tooth extraction* OR teeth extraction* OR dental extraction* OR tooth removal OR teeth removal OR tooth loss OR molar extraction* OR serial extraction) OR AB (tooth extraction* OR teeth extraction* OR dental extraction* OR tooth removal OR teeth removal OR tooth loss OR molar extraction* OR serial extraction) | 355 |

**Search 2 Extraction - Refusal**

PubMed History and Search Details November 6^th^ 2020

| **Search** | **Query** | **Results** |
| --- | --- | --- |
| #5 | #4 NOT (("Adolescent"[Mesh] OR "Child"[Mesh] OR "Infant"[Mesh] OR adolescen*[tiab] OR child*[tiab] OR schoolchild*[tiab] OR infant*[tiab] OR girl*[tiab] OR boy[tiab] OR boys[tiab] OR boyhood[tiab] OR teen[tiab] OR teens[tiab] OR teenager*[tiab] OR youth*[tiab] OR pediatr*[tiab] OR paediatr*[tiab] OR puber*[tiab]) NOT ("Adult"[Mesh] OR adult*[tiab] OR man[tiab] OR men[tiab] OR woman[tiab] OR women[tiab])) | 395 |
| #4 | (#1 OR #2) AND #3 | 486 |
| #3 | "Ethics, Dental"[Mesh] OR "Refusal to Treat"[Mesh] OR "Clinical Decision-Making"[Mesh] OR "Refusal to Treat"[tiab] OR "patient abandonment"[tiab] OR "patient dumping"[tiab] OR "rejection"[tiab] OR "dental ethic*"[tiab] OR "decision making"[tiab] OR "request*"[tiab] | 300,758 |
| #2 | "Dentition, Permanent"[Mesh] OR "permanent teeth"[tiab] OR "secondary teeth"[tiab] OR "secondary tooth"[tiab] OR "adult teeth"[tiab] OR "adult tooth"[tiab] OR "dentes permanent*"[tiab] OR "permanent dentition"[tiab] OR "adult dentition"[tiab] OR "secondary dentition"[tiab] | 9,831 |
| #1 | "Tooth Extraction"[Mesh] OR "tooth extraction*"[tiab] OR "teeth extraction*"[tiab] OR "dental extraction*"[tiab] OR "tooth removal"[tiab] OR "teeth removal"[tiab] OR "tooth loss"[tiab] OR "molar extraction*"[tiab] OR "serial extraction"[tiab] | 28,014 |

Embase History and Search Details November 6^th^ 2020

| **Search** | **Query** | **Results** |
| --- | --- | --- |
| #7 | #6 AND ([article]/lim OR [article in press]/lim OR [editorial]/lim OR [letter]/lim OR [review]/lim OR [short survey]/lim) | 621 |
| #6 | #5 NOT (('adolescent'/exp OR 'child'/exp OR adolescent*:ti,ab OR child*:ti,ab OR schoolchild*:ti,ab OR infant*:ti,ab OR girl*:ti,ab OR boy*:ti,ab OR teen:ti,ab OR teens:ti,ab OR teenager*:ti,ab OR youth*:ti,ab OR pediatr*:ti,ab OR paediatr*:ti,ab OR puber*:ti,ab) NOT ('adult'/exp OR 'aged'/exp OR 'middle aged'/exp OR adult*:ti,ab OR man:ti,ab OR men:ti,ab OR woman:ti,ab OR women:ti,ab)) | 606 |
| #5 | #3 AND #4 | 735 |
| #4 | #1 OR #2 | 41,504 |
| #3 | 'medical ethics'/exp OR 'patient abandonment'/exp OR 'Refusal to Treat':ti,ab OR 'clinical decision making'/de OR 'patient abandonment':ti,ab OR 'patient dumping':ti,ab OR rejection:ti,ab OR 'dental ethic*':ti,ab OR 'decision making':ti,ab OR request*:ti,ab | 642,351 |
| #2 | 'secondary dentition'/exp OR 'permanent teeth':ti,ab OR 'secondary teeth':ti,ab OR 'secondary tooth':ti,ab OR 'adult teeth':ti,ab OR 'adult tooth':ti,ab OR 'dentes permanent*':ti,ab OR 'permanent dentition':ti,ab OR 'adult dentition':ti,ab OR 'secondary dentition':ti,ab | 9,519 |
| #1 | 'tooth extraction'/exp OR 'tooth extraction*':ti,ab OR 'teeth extraction*':ti,ab OR 'dental extraction*':ti,ab OR 'tooth removal':ti,ab OR 'teeth removal':ti,ab OR 'tooth loss':ti,ab OR 'molar extraction*':ti,ab OR 'serial extraction':ti,ab | 32,747 |

APA PsycInfo Refusal History and Search Details November 6^th^ 2020

| **Search** | **Query** | **Results** |
| --- | --- | --- |
| S5 | S4 NOT ((ZG ("adolescence (13-17 yrs)" OR "childhood (birth-12 yrs)" OR "infancy (2-23 mo)” OR "neonatal (birth-1 mo)" OR "preschool age (2-5 yrs)" OR "school age (6-12 yrs)") OR TI (adolescen* OR child* OR schoolchild* OR infant* OR girl* OR boy* OR teen OR teens OR teenager* OR youth* OR pediatr* OR paediatr* OR puber*) OR AB (adolescen* OR child* OR schoolchild* OR infant* OR girl* OR boy* OR teen OR teens OR teenager* OR youth* OR pediatr* OR paediatr* OR puber*)) NOT (ZG ("adulthood (18 yrs & older)" OR "aged (65 yrs & older)" OR "middle age (40-64 yrs)" OR "thirties (30-39 yrs)" OR "very old (85 yrs & older)") OR TI (adult* OR man OR men OR woman OR women) OR AB (adult* OR man OR men OR woman OR women))) | 7 |
| S4 | (S1 OR S2) AND S3 | 7 |
| S3 | DE "Bioethics" OR TI ("patient abandonment" OR "Refusal to Treat" OR "clinical decision making" OR "patient dumping" OR rejection OR "dental ethic*" OR "decision making" OR request*) OR AB ("patient abandonment" OR "Refusal to Treat" OR "clinical decision making" OR "patient dumping" OR rejection OR "dental ethic*" OR "decision making" OR request*) | 130,098 |
| S2 | TI ("permanent teeth" OR "secondary teeth" OR "secondary tooth" OR "adult teeth" OR "adult tooth" OR "dentes permanents" OR "permanent dentition" OR "adult dentition" OR "secondary dentition") OR AB ("permanent teeth" OR "secondary teeth" OR "secondary tooth" OR "adult teeth" OR "adult tooth" OR "dentes permanents" OR "permanent dentition" OR "adult dentition" OR "secondary dentition") | 65 |
| S1 | TI (tooth extraction* OR teeth extraction* OR dental extraction* OR tooth removal OR teeth removal OR tooth loss OR molar extraction* OR serial extraction) OR AB (tooth extraction* OR teeth extraction* OR dental extraction* OR tooth removal OR teeth removal OR tooth loss OR molar extraction* OR serial extraction) | 355 |
